# Bis(3,5-dimethyl­pyridine-κ*N*)bis­(tri-*tert*-butoxy­silanethiol­ato-κ*S*)chromium(II) toluene solvate

**DOI:** 10.1107/S1600536809021680

**Published:** 2009-06-13

**Authors:** Anna Ciborska, Katarzyna Baranowska, Wiesław Wojnowski

**Affiliations:** a Department of Chemistry, Technical University of Gdańsk, 11/12 G. Narutowicz St., 80233–PL Gdańsk, Poland

## Abstract

In the title chromium silanethiol­ate, [Cr(C_12_H_27_O_3_SSi)_2_(C_7_H_9_N)_2_]·C_7_H_8_, the Cr^II^ atom is coordinated by two S and two N atoms in a distorted square-planar geometrical arrangement. The mononuclear mol­ecule lies on a twofold axis that passes through the pyridine N atoms. The toluene solvent mol­ecule is equally disordered about a twofold axis.

## Related literature

For the synthetic procedures, see: Perrin & Armarego (1988 ▶); Piękoś & Wojnowski (1962 ▶); Wojnowska & Wojnowski (1974 ▶). For the use of such complexes in model studies of proteins, see: Becker *et al.* (2002 ▶); Dołęga *et al.* (2008 ▶). For another Cr–thiol­ate, see: Dorfman *et al.* (1985 ▶). For related strutures, see: Ciborska *et al.* (2007 ▶, 2008 ▶).
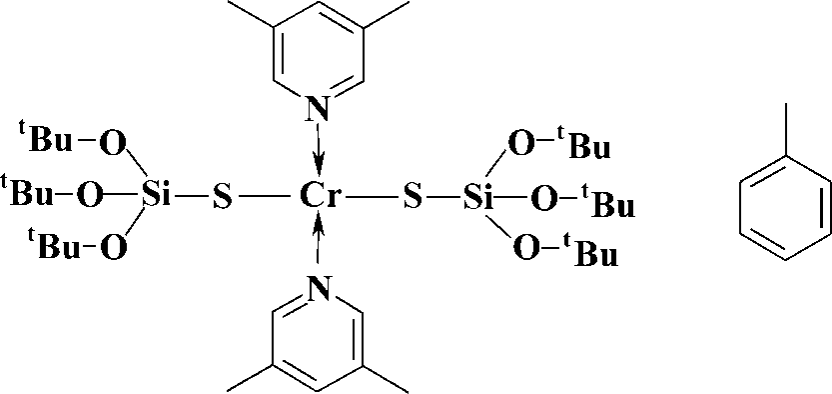


## Experimental

### 

#### Crystal data

[Cr(C_12_H_27_O_3_SSi)_2_(C_7_H_9_N)_2_]·C_7_H_8_
*M* *_r_* = 917.41
Monoclinic, 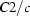

*a* = 19.6147 (4) Å
*b* = 17.1521 (17) Å
*c* = 17.2221 (9) Å
β = 112.047 (5)°
*V* = 5370.4 (6) Å^3^
*Z* = 4
Mo *K*α radiation
μ = 0.38 mm^−1^
*T* = 120 K
0.32 × 0.30 × 0.19 mm

#### Data collection

Oxford Diffraction KM-4-CCD diffractometer
Absorption correction: none
18436 measured reflections
5260 independent reflections
4788 reflections with *I* > 2σ(*I*)
*R*_int_ = 0.031

#### Refinement

*R*[*F* ^2^ > 2σ(*F* ^2^)] = 0.048
*wR*(*F* ^2^) = 0.148
*S* = 1.11
5260 reflections
296 parameters
1 restraint
H-atom parameters constrained
Δρ_max_ = 1.14 e Å^−3^
Δρ_min_ = −0.78 e Å^−3^

### 

Data collection: *CrysAlis CCD* (Oxford Diffraction, 2006 ▶); cell refinement: *CrysAlis RED* (Oxford Diffraction, 2006 ▶); data reduction: *CrysAlis RED*; program(s) used to solve structure: *SHELXS97* (Sheldrick, 2008 ▶); program(s) used to refine structure: *SHELXL97* (Sheldrick, 2008 ▶); molecular graphics: *ORTEP-3 for Windows* (Farrugia, 1997 ▶); software used to prepare material for publication: *WinGX* (Farrugia, 1999 ▶).

## Supplementary Material

Crystal structure: contains datablocks global, I. DOI: 10.1107/S1600536809021680/ng2589sup1.cif

Structure factors: contains datablocks I. DOI: 10.1107/S1600536809021680/ng2589Isup2.hkl

Additional supplementary materials:  crystallographic information; 3D view; checkCIF report

## Figures and Tables

**Table 1:** Selected bond lengths (Å)

Cr1—N1	2.136 (3)
Cr1—N2	2.153 (3)
Cr1—S1	2.4426 (6)
S1—Si1	2.0694 (8)
Si1—O3	1.6342 (17)
Si1—O2	1.6370 (17)
Si1—O1	1.6480 (17)

**Fig. 1.**
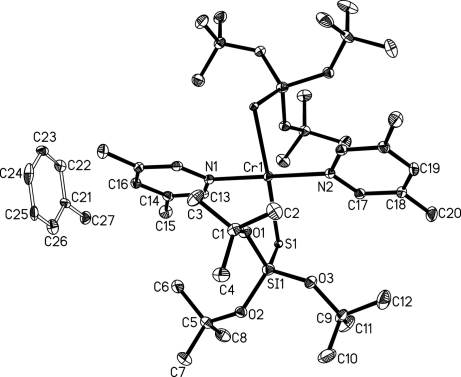
A view of the molecule of (I), showing the atom-labelling scheme. Displacement ellipsoids are drawn at the 30% probability level and H atoms have been omitted.

## supplementary crystallographic information

### Comment

The large development of transition-metal silanethiolate chemistry results from its potential to form new types of complexes with interesting chemical properties. These complexes may be used in model studies on structural and catalytic metal centers in proteins (Becker *et al.* 2002; Dołęga *et al.* 2008). Here we present the synthesis and molecular structure of the chromium(II), tri-*tert*-butoxysilanethiolate complex [Cr(C_12_H_27_O_3_SSi)_2_(C_7_H_9_N)_2_] C_7_H_8_.The crystal structure of the title compound (I) is one of the few structurally defined four-coordinate Cr^II^ thiolate complexes (Dorfman *et al.* 1985; Ciborska *et al.* 2008). This complex was obtained as light-blue crystals in the reaction of anhydrous Cr^II^ chloride with sodium tri-*tert*-butoxysilanethiolate and 3,5-dimethylpyridine. The Cr^II^ ion is coordinated by two S atoms from the tri-*tert*-butoxysilanethiolate ligands and two N atoms from the 3,5-dimethylpyridine molecules. The *trans* angles of the square base are then described by S—Cr—S and N—Cr—N, which are very close to 180°. The Cr—S bond lengths in (I) are very similar to the corresponding values of *ca* 2.4 Å observed in the other silanethiolates (Ciborska *et al.*2007). The Cr—N bond lengths are like these found in the [Cr(C_12_H_27_O_3_SSi)_2_(C_6_H_15_N)_2_]. Selected data of important bond lengths and angles are compared in Table 1.

### Experimental

The synthesis was carried out under an atmosphere of nitrogen, using standard Schlenk techniques. Solvents and the amine were purified and dried by standard methods (Perrin & Armarego, 1988). The substrate (*^t^*BuO)_3_SiSNa was prepared according to literature methods (Piękoś & Wojnowski, 1962; Wojnowska & Wojnowski, 1974). The title compound was synthesized by addition of the CrCl_2_ solution (0.143 g, 1.16 mmol) in tetrahydrofuran (10 ml) to (*^t^*BuO)_3_SiSNa solution (0.833 g, 2.7 mmol) in toluene (10 ml) and stirring for 1 h.

3,5-Dimethylpyridine (0,267 g, 0.28 ml, 2.5 mmol) was subsequently added to the solution and stirred for next 12 h. After that the mixture was concentrated and cooled (250 K) to afford light-blue crystals.

### Refinement

All C–H hydrogen atoms were refined as riding on carbon atoms with methyl C–H = 0.98 Å, aromatic C–H = 0.95 Å and *U*_ĩso_(H)=1.2 *U*_eq_(C) for aromatic CH and 1.5*U*_eq_(C) for methyl groups.

The toluene molecule was allowed to refine off the twofold axis.  The aromatic ring was refined as a rigid hexagon of 1.39 Å sides.  The phenyl–methyl distance was restrained to 1.50±0.01 Å.

### Crystal data

**Table:**

[Cr(C_12_H_27_O_3_SSi)_2_(C_7_H_9_N)_2_]·C_7_H_8_	*F*(000) = 1984
*M**_r_* = 917.41	*D*_x_ = 1.135 Mg m^−^^3^
Monoclinic, *C*2/*c*	Mo *K*α radiation, λ = 0.71073 Å
Hall symbol: -C 2yc	Cell parameters from 20255 reflections
*a* = 19.6147 (4) Å	θ = 2.4–32.5°
*b* = 17.1521 (17) Å	µ = 0.38 mm^−^^1^
*c* = 17.2221 (9) Å	*T* = 120 K
β = 112.047 (5)°	Prism, blue
*V* = 5370.4 (6) Å^3^	0.32 × 0.3 × 0.19 mm
*Z* = 4	

### Data collection

**Table:**

Oxford Diffraction KM-4-CCD diffractometer	4788 reflections with *I* > 2σ(*I*)
graphite	*R*_int_ = 0.031
Detector resolution: 8.1883 pixels mm^-1^	θ_max_ = 26°, θ_min_ = 2.7°
ω scans	*h* = −24→24
18436 measured reflections	*k* = −15→21
5260 independent reflections	*l* = −21→21

### Refinement

**Table:**

Refinement on *F*^2^	Primary atom site location: structure-invariant direct methods
Least-squares matrix: full	Secondary atom site location: difference Fourier map
*R*[*F*^2^ > 2σ(*F*^2^)] = 0.048	Hydrogen site location: inferred from neighbouring sites
*wR*(*F*^2^) = 0.148	H-atom parameters constrained
*S* = 1.11	*w* = 1/[σ^2^(*F*_o_^2^) + (0.0822*P*)^2^ + 9.051*P*] where *P* = (*F*_o_^2^ + 2*F*_c_^2^)/3
5260 reflections	(Δ/σ)_max_ < 0.001
296 parameters	Δρ_max_ = 1.14 e Å^−^^3^
1 restraint	Δρ_min_ = −0.78 e Å^−^^3^

### Special details

**Table:**

Geometry. All s.u.'s (except the s.u. in the dihedral angle between two l.s. planes) are estimated using the full covariance matrix. The cell s.u.'s are taken into account individually in the estimation of s.u.'s in distances, angles and torsion angles; correlations between s.u.'s in cell parameters are only used when they are defined by crystal symmetry. An approximate (isotropic) treatment of cell s.u.'s is used for estimating s.u.'s involving l.s. planes.
Refinement. Refinement of *F*^2^ against ALL reflections. The weighted *R*-factor *wR* and goodness of fit *S* are based on *F*^2^, conventional *R*-factors *R* are based on *F*, with *F* set to zero for negative *F*^2^. The threshold expression of *F*^2^ > 2σ(*F*^2^) is used only for calculating *R*-factors(gt) *etc*. and is not relevant to the choice of reflections for refinement. *R*-factors based on *F*^2^ are statistically about twice as large as those based on *F*, and *R*- factors based on ALL data will be even larger.

### Fractional atomic coordinates and isotropic or equivalent isotropic displacement parameters (Å^2^)

**Table:**

	*x*	*y*	*z*	*U*_iso_*/*U*_eq_	Occ. (<1)
Cr1	0	0.01937 (3)	0.25	0.01892 (16)	
S1	0.10684 (3)	0.02265 (3)	0.38160 (3)	0.01235 (16)	
Si1	0.17518 (3)	0.00414 (4)	0.31651 (4)	0.02545 (17)	
O1	0.12513 (9)	0.02386 (9)	0.21779 (10)	0.0220 (4)	
O2	0.25116 (9)	0.05527 (10)	0.35177 (10)	0.0244 (4)	
O3	0.20219 (9)	−0.08576 (9)	0.31481 (10)	0.0244 (4)	
N1	0	0.14391 (15)	0.25	0.0205 (6)	
N2	0	−0.10614 (15)	0.25	0.0200 (5)	
C1	0.14053 (14)	0.01078 (14)	0.14221 (14)	0.0240 (5)	
C2	0.11660 (17)	−0.07146 (16)	0.11088 (17)	0.0351 (6)	
H2A	0.1434	−0.1091	0.1545	0.053*	
H2B	0.1273	−0.0813	0.0605	0.053*	
H2C	0.0637	−0.0771	0.0974	0.053*	
C3	0.09435 (17)	0.07101 (17)	0.07930 (16)	0.0364 (6)	
H3A	0.0422	0.063	0.0692	0.055*	
H3B	0.102	0.0652	0.0265	0.055*	
H3C	0.1091	0.1235	0.1018	0.055*	
C4	0.22130 (16)	0.02209 (17)	0.15862 (17)	0.0346 (6)	
H4A	0.2354	0.076	0.1763	0.052*	
H4B	0.2301	0.0113	0.1073	0.052*	
H4C	0.2507	−0.0137	0.2029	0.052*	
C5	0.26593 (14)	0.13476 (14)	0.38092 (16)	0.0274 (5)	
C6	0.20424 (15)	0.18905 (15)	0.32786 (19)	0.0364 (6)	
H6A	0.1976	0.184	0.2688	0.055*	
H6B	0.2171	0.243	0.346	0.055*	
H6C	0.1584	0.1749	0.3347	0.055*	
C7	0.33779 (15)	0.15658 (17)	0.37182 (18)	0.0356 (6)	
H7A	0.3762	0.1193	0.403	0.053*	
H7B	0.3525	0.2092	0.3941	0.053*	
H7C	0.331	0.1553	0.3125	0.053*	
C8	0.27486 (16)	0.13697 (17)	0.47279 (17)	0.0369 (6)	
H8A	0.2282	0.1227	0.4776	0.055*	
H8B	0.2889	0.1897	0.495	0.055*	
H8C	0.3132	0.1	0.5048	0.055*	
C9	0.25415 (14)	−0.13435 (15)	0.37758 (16)	0.0291 (5)	
C10	0.32921 (17)	−0.1211 (2)	0.3753 (2)	0.0541 (9)	
H10A	0.3283	−0.1342	0.3195	0.081*	
H10B	0.3652	−0.1542	0.4173	0.081*	
H10C	0.343	−0.0663	0.3875	0.081*	
C11	0.25326 (19)	−0.11775 (19)	0.46367 (18)	0.0451 (8)	
H11A	0.2685	−0.0637	0.4793	0.068*	
H11B	0.2873	−0.1532	0.5047	0.068*	
H11C	0.2034	−0.1256	0.4626	0.068*	
C12	0.2294 (2)	−0.21788 (17)	0.3515 (2)	0.0490 (8)	
H12A	0.1813	−0.2267	0.3552	0.073*	
H12B	0.2654	−0.2543	0.3888	0.073*	
H12C	0.2255	−0.2264	0.2937	0.073*	
C13	0.00905 (12)	0.18436 (13)	0.31980 (14)	0.0220 (5)	
H13	0.0157	0.1562	0.3696	0.026*	
C14	0.00919 (13)	0.26521 (14)	0.32324 (15)	0.0234 (5)	
C15	0.02036 (16)	0.30602 (16)	0.40425 (17)	0.0352 (6)	
H15A	0.0273	0.2672	0.4484	0.053*	
H15B	−0.0229	0.338	0.3975	0.053*	
H15C	0.064	0.3394	0.4197	0.053*	
C16	0	0.30546 (19)	0.25	0.0254 (7)	
H16	0	0.3608	0.25	0.03*	
C17	0.02288 (12)	−0.14715 (13)	0.32193 (14)	0.0217 (5)	
H17	0.0402	−0.1192	0.3733	0.026*	
C18	0.02260 (13)	−0.22816 (14)	0.32521 (15)	0.0250 (5)	
C19	0	−0.26836 (19)	0.25	0.0270 (7)	
H19	0	−0.3237	0.25	0.032*	
C20	0.04651 (18)	−0.26906 (16)	0.40847 (17)	0.0371 (6)	
H20A	0.0038	−0.2936	0.4149	0.056*	
H20B	0.0683	−0.2311	0.4536	0.056*	
H20C	0.083	−0.3091	0.4112	0.056*	
C21	0.0088 (6)	0.5182 (7)	0.3429 (6)	0.0241 (15)	0.5
C22	−0.0610 (6)	0.5206 (13)	0.2802 (6)	0.028 (2)	0.5
H22	−0.1032	0.5226	0.2946	0.034*	0.5
C23	−0.0690 (8)	0.5202 (13)	0.1965 (6)	0.029 (2)	0.5
H23	−0.1167	0.5218	0.1537	0.035*	0.5
C24	−0.0072 (9)	0.5173 (8)	0.1755 (6)	0.048 (4)	0.5
H24	−0.0126	0.517	0.1183	0.057*	0.5
C25	0.0627 (8)	0.5149 (14)	0.2382 (7)	0.036 (3)	0.5
H25	0.1049	0.513	0.2239	0.044*	0.5
C26	0.0706 (7)	0.5154 (13)	0.3219 (7)	0.039 (3)	0.5
H26	0.1184	0.5137	0.3648	0.047*	0.5
C27	0.0179 (4)	0.5168 (3)	0.4338 (4)	0.0368 (13)	0.5
H27A	0.0156	0.5702	0.453	0.055*	0.5
H27B	0.0656	0.4937	0.4674	0.055*	0.5
H27C	−0.0216	0.4857	0.4401	0.055*	0.5

### Atomic displacement parameters (Å^2^)

**Table:**

	*U*^11^	*U*^22^	*U*^33^	*U*^12^	*U*^13^	*U*^23^
Cr1	0.0213 (3)	0.0152 (3)	0.0155 (3)	0	0.0016 (2)	0
S1	0.0139 (3)	0.0144 (3)	0.0075 (3)	−0.0006 (2)	0.0026 (2)	−0.00006 (19)
Si1	0.0257 (3)	0.0285 (3)	0.0204 (3)	0.0003 (2)	0.0066 (2)	0.0009 (2)
O1	0.0229 (9)	0.0295 (9)	0.0131 (8)	0.0027 (6)	0.0061 (7)	0.0011 (6)
O2	0.0225 (8)	0.0255 (9)	0.0236 (8)	−0.0022 (7)	0.0068 (7)	−0.0024 (7)
O3	0.0275 (9)	0.0237 (8)	0.0200 (8)	0.0046 (7)	0.0067 (7)	0.0016 (6)
N1	0.0193 (13)	0.0177 (12)	0.0214 (14)	0	0.0041 (11)	0
N2	0.0192 (13)	0.0183 (13)	0.0207 (13)	0	0.0055 (11)	0
C1	0.0275 (13)	0.0318 (13)	0.0137 (11)	0.0027 (10)	0.0088 (10)	0.0008 (9)
C2	0.0471 (16)	0.0386 (14)	0.0239 (13)	−0.0075 (12)	0.0181 (12)	−0.0066 (11)
C3	0.0457 (16)	0.0467 (16)	0.0183 (12)	0.0147 (13)	0.0136 (11)	0.0066 (11)
C4	0.0306 (14)	0.0506 (17)	0.0263 (13)	−0.0019 (12)	0.0150 (11)	0.0005 (11)
C5	0.0252 (12)	0.0269 (12)	0.0288 (13)	−0.0064 (10)	0.0087 (10)	−0.0043 (10)
C6	0.0303 (14)	0.0275 (13)	0.0488 (17)	−0.0019 (11)	0.0118 (13)	0.0023 (12)
C7	0.0284 (14)	0.0377 (15)	0.0408 (16)	−0.0096 (11)	0.0130 (12)	−0.0052 (12)
C8	0.0353 (15)	0.0412 (15)	0.0335 (15)	−0.0105 (12)	0.0121 (12)	−0.0136 (12)
C9	0.0285 (13)	0.0301 (13)	0.0300 (13)	0.0100 (10)	0.0125 (11)	0.0108 (10)
C10	0.0298 (16)	0.072 (2)	0.060 (2)	0.0130 (15)	0.0159 (15)	0.0320 (18)
C11	0.0519 (18)	0.0530 (18)	0.0287 (15)	0.0221 (15)	0.0133 (13)	0.0149 (13)
C12	0.059 (2)	0.0293 (15)	0.063 (2)	0.0131 (14)	0.0279 (17)	0.0060 (14)
C13	0.0190 (11)	0.0231 (11)	0.0215 (11)	−0.0005 (9)	0.0048 (9)	0.0004 (9)
C14	0.0196 (11)	0.0231 (11)	0.0267 (12)	−0.0001 (9)	0.0076 (9)	−0.0039 (9)
C15	0.0408 (15)	0.0310 (13)	0.0333 (14)	0.0023 (11)	0.0135 (12)	−0.0091 (11)
C16	0.0237 (17)	0.0175 (15)	0.0328 (19)	0	0.0082 (14)	0
C17	0.0206 (11)	0.0227 (11)	0.0199 (11)	−0.0007 (9)	0.0054 (9)	0.0005 (9)
C18	0.0240 (12)	0.0216 (11)	0.0275 (13)	0.0002 (9)	0.0073 (10)	0.0048 (9)
C19	0.0297 (18)	0.0162 (15)	0.0338 (19)	0	0.0105 (15)	0
C20	0.0503 (17)	0.0275 (13)	0.0287 (14)	−0.0016 (12)	0.0094 (12)	0.0078 (11)
C21	0.020 (4)	0.014 (3)	0.034 (4)	−0.001 (2)	0.006 (3)	−0.003 (3)
C22	0.031 (5)	0.018 (4)	0.034 (4)	0.001 (3)	0.011 (4)	−0.008 (4)
C23	0.025 (4)	0.022 (4)	0.032 (5)	−0.002 (3)	0.001 (3)	−0.006 (4)
C24	0.090 (10)	0.026 (5)	0.038 (6)	0.000 (5)	0.036 (6)	0.003 (4)
C25	0.039 (5)	0.027 (5)	0.050 (7)	0.003 (4)	0.026 (4)	0.012 (6)
C26	0.045 (6)	0.027 (6)	0.047 (6)	−0.001 (4)	0.018 (6)	0.003 (6)
C27	0.045 (3)	0.024 (3)	0.038 (3)	0.000 (2)	0.012 (3)	0.000 (2)

### Geometric parameters (Å, °)

**Table:**

Cr1—N1	2.136 (3)	C10—H10A	0.98
Cr1—N2	2.153 (3)	C10—H10B	0.98
Cr1—S1^i^	2.4426 (6)	C10—H10C	0.98
Cr1—S1	2.4426 (6)	C11—H11A	0.98
S1—Si1	2.0694 (8)	C11—H11B	0.98
Si1—O3	1.6342 (17)	C11—H11C	0.98
Si1—O2	1.6370 (17)	C12—H12A	0.98
Si1—O1	1.6480 (17)	C12—H12B	0.98
O1—C1	1.460 (3)	C12—H12C	0.98
O2—C5	1.444 (3)	C13—C14	1.388 (3)
O3—C9	1.441 (3)	C13—H13	0.95
N1—C13	1.341 (3)	C14—C16	1.389 (3)
N1—C13^i^	1.341 (3)	C14—C15	1.502 (3)
N2—C17^i^	1.347 (3)	C15—H15A	0.98
N2—C17	1.347 (3)	C15—H15B	0.98
C1—C4	1.514 (4)	C15—H15C	0.98
C1—C2	1.520 (4)	C16—C14^i^	1.389 (3)
C1—C3	1.525 (3)	C16—H16	0.95
C2—H2A	0.98	C17—C18	1.391 (3)
C2—H2B	0.98	C17—H17	0.95
C2—H2C	0.98	C18—C19	1.385 (3)
C3—H3A	0.98	C18—C20	1.504 (3)
C3—H3B	0.98	C19—C18^i^	1.385 (3)
C3—H3C	0.98	C19—H19	0.95
C4—H4A	0.98	C20—H20A	0.98
C4—H4B	0.98	C20—H20B	0.98
C4—H4C	0.98	C20—H20C	0.98
C5—C7	1.522 (4)	C21—C22	1.39
C5—C8	1.526 (4)	C21—C26	1.39
C5—C6	1.528 (4)	C21—C27	1.508 (9)
C6—H6A	0.98	C22—C23	1.39
C6—H6B	0.98	C22—H22	0.95
C6—H6C	0.98	C23—C24	1.39
C7—H7A	0.98	C23—H23	0.95
C7—H7B	0.98	C24—C25	1.39
C7—H7C	0.98	C24—H24	0.95
C8—H8A	0.98	C25—C26	1.39
C8—H8B	0.98	C25—H25	0.95
C8—H8C	0.98	C26—H26	0.95
C9—C10	1.505 (4)	C27—H27A	0.98
C9—C11	1.516 (4)	C27—H27B	0.98
C9—C12	1.525 (4)	C27—H27C	0.98
			
N1—Cr1—N2	180	O3—C9—C11	111.1 (2)
N1—Cr1—S1^i^	88.677 (16)	C10—C9—C11	111.6 (3)
N2—Cr1—S1^i^	91.323 (16)	O3—C9—C12	105.4 (2)
N1—Cr1—S1	88.677 (16)	C10—C9—C12	109.9 (3)
N2—Cr1—S1	91.323 (16)	C11—C9—C12	110.2 (2)
S1^i^—Cr1—S1	177.35 (3)	C9—C10—H10A	109.5
Si1—S1—Cr1	89.91 (3)	C9—C10—H10B	109.5
O3—Si1—O2	104.84 (9)	H10A—C10—H10B	109.5
O3—Si1—O1	104.28 (9)	C9—C10—H10C	109.5
O2—Si1—O1	112.30 (9)	H10A—C10—H10C	109.5
O3—Si1—S1	115.77 (7)	H10B—C10—H10C	109.5
O2—Si1—S1	113.67 (7)	C9—C11—H11A	109.5
O1—Si1—S1	105.74 (7)	C9—C11—H11B	109.5
C1—O1—Si1	130.23 (15)	H11A—C11—H11B	109.5
C5—O2—Si1	132.12 (15)	C9—C11—H11C	109.5
C9—O3—Si1	132.47 (16)	H11A—C11—H11C	109.5
C13—N1—C13^i^	117.7 (3)	H11B—C11—H11C	109.5
C13—N1—Cr1	121.16 (14)	C9—C12—H12A	109.5
C13^i^—N1—Cr1	121.16 (14)	C9—C12—H12B	109.5
C17^i^—N2—C17	117.0 (3)	H12A—C12—H12B	109.5
C17^i^—N2—Cr1	121.49 (14)	C9—C12—H12C	109.5
C17—N2—Cr1	121.49 (14)	H12A—C12—H12C	109.5
O1—C1—C4	111.5 (2)	H12B—C12—H12C	109.5
O1—C1—C2	108.63 (19)	N1—C13—C14	123.6 (2)
C4—C1—C2	110.3 (2)	N1—C13—H13	118.2
O1—C1—C3	105.24 (19)	C14—C13—H13	118.2
C4—C1—C3	110.3 (2)	C13—C14—C16	117.4 (2)
C2—C1—C3	110.8 (2)	C13—C14—C15	120.2 (2)
C1—C2—H2A	109.5	C16—C14—C15	122.4 (2)
C1—C2—H2B	109.5	C14—C15—H15A	109.5
H2A—C2—H2B	109.5	C14—C15—H15B	109.5
C1—C2—H2C	109.5	H15A—C15—H15B	109.5
H2A—C2—H2C	109.5	C14—C15—H15C	109.5
H2B—C2—H2C	109.5	H15A—C15—H15C	109.5
C1—C3—H3A	109.5	H15B—C15—H15C	109.5
C1—C3—H3B	109.5	C14^i^—C16—C14	120.4 (3)
H3A—C3—H3B	109.5	C14^i^—C16—H16	119.8
C1—C3—H3C	109.5	C14—C16—H16	119.8
H3A—C3—H3C	109.5	N2—C17—C18	123.7 (2)
H3B—C3—H3C	109.5	N2—C17—H17	118.2
C1—C4—H4A	109.5	C18—C17—H17	118.2
C1—C4—H4B	109.5	C19—C18—C17	117.6 (2)
H4A—C4—H4B	109.5	C19—C18—C20	122.3 (2)
C1—C4—H4C	109.5	C17—C18—C20	120.0 (2)
H4A—C4—H4C	109.5	C18—C19—C18^i^	120.3 (3)
H4B—C4—H4C	109.5	C18—C19—H19	119.9
O2—C5—C7	105.6 (2)	C18^i^—C19—H19	119.9
O2—C5—C8	108.3 (2)	C18—C20—H20A	109.5
C7—C5—C8	110.5 (2)	C18—C20—H20B	109.5
O2—C5—C6	110.9 (2)	H20A—C20—H20B	109.5
C7—C5—C6	110.2 (2)	C18—C20—H20C	109.5
C8—C5—C6	111.2 (2)	H20A—C20—H20C	109.5
C5—C6—H6A	109.5	H20B—C20—H20C	109.5
C5—C6—H6B	109.5	C22—C21—C26	120
H6A—C6—H6B	109.5	C22—C21—C27	120.3 (4)
C5—C6—H6C	109.5	C26—C21—C27	119.7 (4)
H6A—C6—H6C	109.5	C21—C22—C23	120
H6B—C6—H6C	109.5	C21—C22—H22	120
C5—C7—H7A	109.5	C23—C22—H22	120
C5—C7—H7B	109.5	C24—C23—C22	120
H7A—C7—H7B	109.5	C24—C23—H23	120
C5—C7—H7C	109.5	C22—C23—H23	120
H7A—C7—H7C	109.5	C23—C24—C25	120
H7B—C7—H7C	109.5	C23—C24—H24	120
C5—C8—H8A	109.5	C25—C24—H24	120
C5—C8—H8B	109.5	C26—C25—C24	120
H8A—C8—H8B	109.5	C26—C25—H25	120
C5—C8—H8C	109.5	C24—C25—H25	120
H8A—C8—H8C	109.5	C25—C26—C21	120
H8B—C8—H8C	109.5	C25—C26—H26	120
O3—C9—C10	108.4 (2)	C21—C26—H26	120
			
N1—Cr1—S1—Si1	−98.83 (2)	Si1—O2—C5—C8	83.6 (3)
N2—Cr1—S1—Si1	81.17 (2)	Si1—O2—C5—C6	−38.7 (3)
Cr1—S1—Si1—O3	−98.51 (7)	Si1—O3—C9—C10	−87.7 (3)
Cr1—S1—Si1—O2	140.02 (7)	Si1—O3—C9—C11	35.3 (3)
Cr1—S1—Si1—O1	16.37 (7)	Si1—O3—C9—C12	154.7 (2)
O3—Si1—O1—C1	−47.8 (2)	C13^i^—N1—C13—C14	0.29 (17)
O2—Si1—O1—C1	65.2 (2)	Cr1—N1—C13—C14	−179.71 (17)
S1—Si1—O1—C1	−170.29 (18)	N1—C13—C14—C16	−0.6 (3)
O3—Si1—O2—C5	−167.93 (19)	N1—C13—C14—C15	−179.5 (2)
O1—Si1—O2—C5	79.5 (2)	C13—C14—C16—C14^i^	0.26 (15)
S1—Si1—O2—C5	−40.5 (2)	C15—C14—C16—C14^i^	179.1 (3)
O2—Si1—O3—C9	52.9 (2)	C17^i^—N2—C17—C18	−1.29 (17)
O1—Si1—O3—C9	171.1 (2)	Cr1—N2—C17—C18	178.71 (17)
S1—Si1—O3—C9	−73.2 (2)	N2—C17—C18—C19	2.5 (3)
S1^i^—Cr1—N1—C13	135.55 (11)	N2—C17—C18—C20	−177.7 (2)
S1—Cr1—N1—C13	−44.45 (11)	C17—C18—C19—C18^i^	−1.16 (15)
S1^i^—Cr1—N1—C13^i^	−44.45 (11)	C20—C18—C19—C18^i^	179.0 (3)
S1—Cr1—N1—C13^i^	135.55 (11)	C26—C21—C22—C23	0
S1^i^—Cr1—N2—C17^i^	31.46 (11)	C27—C21—C22—C23	−178.6 (12)
S1—Cr1—N2—C17^i^	−148.54 (11)	C21—C22—C23—C24	0
S1^i^—Cr1—N2—C17	−148.54 (11)	C22—C23—C24—C25	0
S1—Cr1—N2—C17	31.46 (11)	C23—C24—C25—C26	0
Si1—O1—C1—C4	−35.4 (3)	C24—C25—C26—C21	0
Si1—O1—C1—C2	86.4 (3)	C22—C21—C26—C25	0
Si1—O1—C1—C3	−154.96 (19)	C27—C21—C26—C25	178.6 (12)
Si1—O2—C5—C7	−158.01 (18)		

Symmetry codes: (i) −*x*, *y*, −*z*+1/2.
